# Human‐induced pluripotent stem cell‐derived cerebral organoid of leukoencephalopathy with vanishing white matter

**DOI:** 10.1111/cns.14079

**Published:** 2023-01-17

**Authors:** Jiong Deng, Jie Zhang, Kai Gao, Ling Zhou, Yuwu Jiang, Jingmin Wang, Ye Wu

**Affiliations:** ^1^ Department of Pediatrics Peking University First Hospital Beijing China

**Keywords:** cerebral organoid, dynamic development, eIF2B, induced pluripotent stem cell, leukoencephalopathy with vanishing white matter

## Abstract

**Introduction:**

Leukoencephalopathy with vanishing white matter (VWM) is a rare autosomal recessive leukoencephalopathy resulting from mutations in *EIF2B1‐5*, which encode subunits of eukaryotic translation initiation factor 2B (eIF2B). Studies have found that eIF2B mutation has a certain influence on embryonic brain development. So far, the effect of the eIF2B mutations on the dynamic process of brain development is not fully understood yet.

**Aims:**

Three‐dimensional brain organoid technology has promoted the study of human nervous system developmental diseases in recent years, providing a potential platform for elucidating the pathological mechanism of neurodevelopmental diseases. In this study, we aimed to investigate the effects of eIF2B mutation on the differentiation and development of different nerve cells during dynamic brain development process using 3D brain organoids.

**Results:**

We constructed eIF2B mutant and wild‐type brain organoid model with induced pluripotent stem cell (iPSC). Compared with the wild type, the mutant brain organoids were significantly smaller, accompanied by increase in apoptosis, which might be resulted from overactivation of unfolded protein response (UPR). Neuronal development was delayed in early stage, but with normal superficial neuronal differentiation in later stage. eIF2B mutations resulted in immature astrocytes with increased expression of GFAPδ, nestin, and αB‐crystallin, and there were increased oligodendrocyte progenitor cells, decreased mature oligodendrocytes, and sparse myelin in mutant cerebral organoids in the later stage.

**Conclusion:**

we constructed the first eIF2B mutant cerebral organoids to explore the dynamic brain development process, which provides a platform for further research on the specific pathogenesis of VWM.

## Graphical abstract

Leukoencephalopathy with vanishing white matter (VWM) is a rare autosomal recessive leukoencephalopathy resulting from mutations in *EIF2B1‐5*. This study generates the first *EIF2B4* and *EIF2B5* mutant brain organoids with human‐induced pluripotent stem cells derived from VWM patients and explores the effects of eIF2B mutations on the dynamic process of brain development from early to later stages, which provides a platform for further research on the specific pathogenesis of VWM.

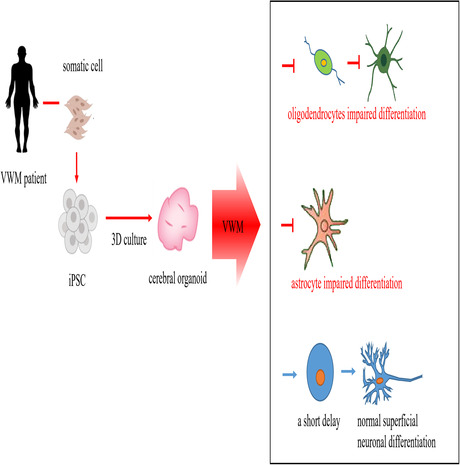

## 1 INTRODUCTION

Leukoencephalopathy with vanishing white matter (VWM) is a genetic disorder due to mutations in genes encoding subunits of eukaryotic translation initiation factor 2B (eIF2B), leading to progressive rarefaction of cerebral white matter. The typical manifestation is progressive motor regression, accompanied by ataxia and epileptic seizures in some patients, and exacerbated by fever or head trauma.^1^, ^2^ We diagnosed the first child with VWM in China in 2007^3^ and have so far diagnosed 60 Chinese children with VWM.^4^ Neuropathology from autopsy samples showed sparseness of deep white matter, increased cystic degeneration without reactive astrocytes proliferation, dysmorphic immature astrocytes, increased oligodendrocytes precursor cells, and foamy oligodendrocytes.^5^ In particular, neuropathological findings from two fetuses with *EIF2B5* gene mutations presented in utero growth retardation and microcephaly with simplified gyral pattern, which indicated the disease might occur from early gestation and may apparent from the second half of pregnancy by a severe impairment of brain development.^6^ So far, the effect of the eIF2B mutations on the dynamic process of brain development is not fully understood yet.

In recent years, the development of 3D brain organoid technology has promoted the study of human nervous system developmental diseases.^7^ It provides a potential platform for elucidating the pathological mechanism of neurodevelopmental diseases. Cerebral organoids have stable phenotypic and genetic characteristics and can be cultured in vitro for a long time to simulate programmed cell growth, space‐specific cell lineage differentiation, and self‐organization during organ development in vivo.^8^, ^9^ Compared with 2D culture environment, 3D organoids have multiple cell types and have tighter cell‐to‐cell connections and interactions, which better mimic organogenesis and pathophysiological characteristics.

In this study, we generated *EIF2B4* and *EIF2B5* mutant brain organoids to explore the effects of eIF2B mutation on the differentiation and development of various neuronal cells during the dynamic development of the brain, which provides new clues for exploring the pathogenic mechanism of VWM.

## 2 METHODS

### 2.1 Generation and maintenance of induced pluripotent stem cells

**FIGURE 1:**
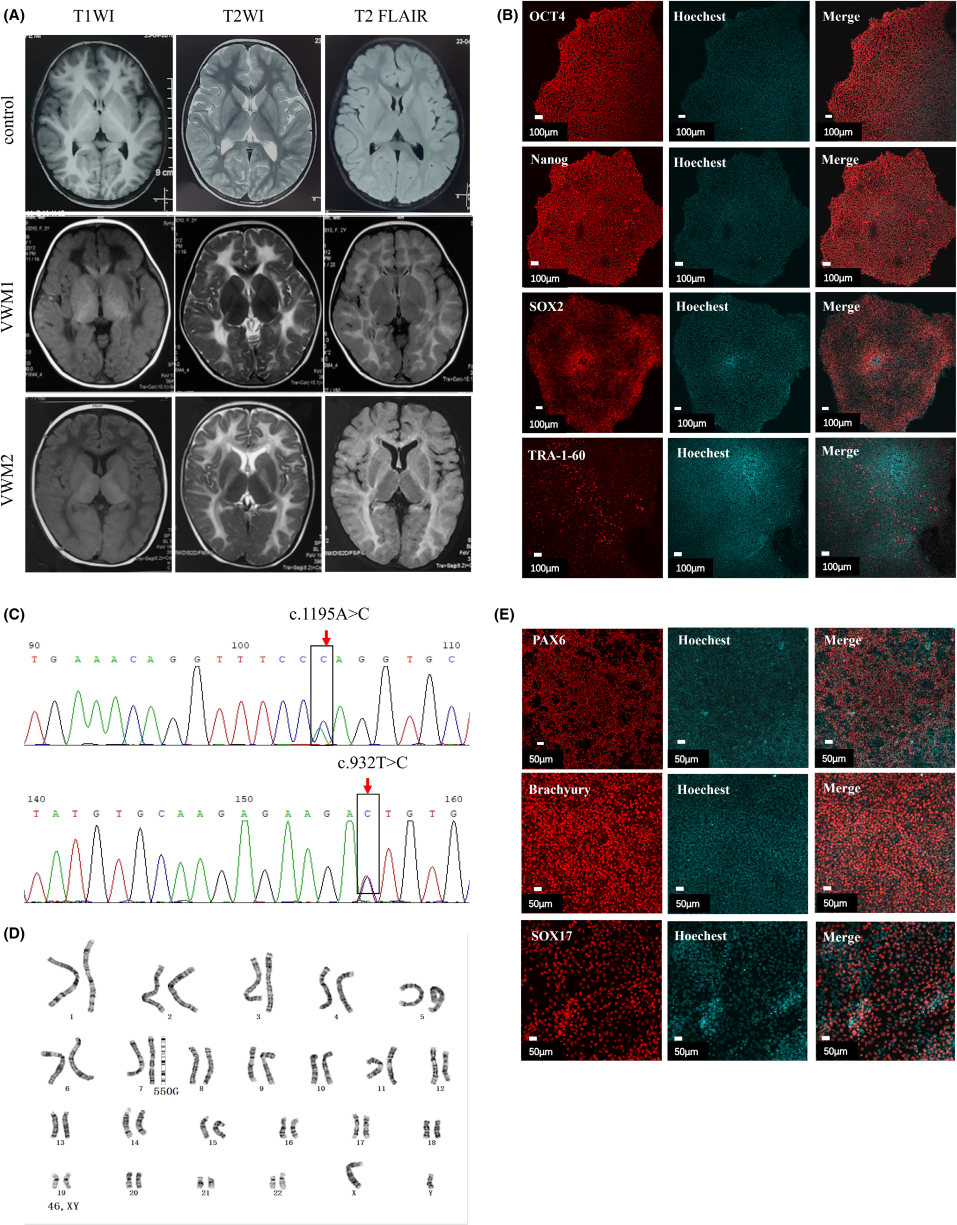
VWM patient‐derived iPSC and identification. (A) Brain MRI of one normal control child (3 years old) and two children with VWM. VWM1 and VWM2 are cases of childhood onset, and the age of onset and MRI age are 12 months and 3 years old, respectively. The white matter of the lesions in children with VWM showed low signal on T1WI, high signal on T2WI, and low signal on T2 FLAIR part (liquefaction sign). The earlier the onset, the more extensive the white matter involvement. (B) Immunofluorescence analysis of pluripotent markers, OCT4, Nanog, SOX2, and TRA‐1‐60, scale bar 100 μm. (C) Sanger generation sequencing analysis of the genomic DNA from VWM2 patient‐derived iPSC showed: *EIF2B4* c.1195A>C; c.932T>C. (D) Karyotype analysis showed 46, XY. (E) Immunofluorescence staining of differential marker: endoderm (SOX17), mesoderm (Brachyury), and ectoderm (PAX6), scale bar 50 μm.

Vanishing white matter patient‐derived iPSCs were obtained by the reprogramming induction of somatic cells from two VWM patients with *EIF2B5* and *EIF2B4* mutations. In addition, the brain MRI showed bilateral, diffuse, and symmetrical involvement of deep white matter, with low signal intensity on TIWI and high signal intensity on T2WI (Figure 1A). VWM1 patient‐derived iPSC was performed as previously reported.^10^ The two patient‐derived iPSC lines were both generated in the same way by neon electroporation with Yamanaka factors (OCT3/4, SOX2, KLF4, and L‐MYC) and Lin28 together (Invitrogen, Epi5 Episomal iPSC Reprogramming Kit, A15960). The iPSC2 qualification was provided in Figure 1B–E. A summary of the clinical information from patients with VWM included in the study can be found in Table 1. Human wild‐type (WT) iPSC was provided by Beijing Cellapy Biotechnology Co. Ltd. All iPSC lines were cultured in dishes coated with matrigel (Corning, 354277) and in mTeSR medium (STEMCELL Technologies, 85850). Cells were passaged using the enzyme‐free Gentle Cell Dissociation Reagent (GCDR, STEMCELL Technologies, 100‐0485), and cell colonies were separated with a pipette tip when cells were 70%–80% confluent.

**TABLE 1:** Clinical information of VWM patients in this study

Categories	VWM1	VWM2
Gender	Male	Male
Onset age	4 years old	12 months
Onset symptom	Regression of motor function	Regression of motor function, epileptic seizures
Outcome	Died at 16 years old	2 years and 7 months old currently, walk alone, actively grasp objects, and has normal cognitive development
Mutated gene	*EIF2B5*	*EIF2B4*
Mutation on genomic DNA	c.1827_1838del(paternal) c.1157G>A(maternal)	c.932T>C(paternal) c.1195A>C(maternal)
Amino acid change	p.610_613del4 p.Gly386Val	p.Ile311Thr p.Lys399Gln

### 2.2 Culture of cerebral organoids

According to a previously reported protocol, cerebral organoids were generated using STEMdiff Cerebral Organoid kit (STEMCELL Technologies, 08570, 08571) following the manufacturers' instructions.^11^ On Day 0, the iPSCs were gently resuspended using GCDR. The cells were centrifuged at 300*g* for 5 min, resuspended in Embryoid Body (EB) Formation medium, and diluted to a concentration of 90,000 cells/ml. Then, 100 μl of the cell suspension was added to each well of a 96‐well round‐bottom ultra‐low attachment plate (Corning, 7007). From Days 2–5, the medium was changed every 2 days. On Days 5–7, EBs were dispensed into each well of a 24‐well ultra‐low attachment plate (Corning, 3473) containing induction medium by using a wide‐bore 200 μl pipette tip. On Days 7–10, the EBs were collected and embedded in Matrigel and cultured in Expansion Medium in a 6‐well ultra‐low attachment plate (Corning, 3471) in suspension culture. On Day 10 and the following days, the plates were transferred to an orbital shaker at a speed of 65 rpm, and the medium was replaced with maturation medium. The medium was changed every 3–4 days. In this study, glial cells were observed up to 20 weeks of culture.

### 2.3 Immunofluorescence analysis

Organoids were fixed with 4% paraformaldehyde solution at 4°C overnight. Then samples were equilibrated in 30% sucrose solution at 4°C overnight. Organoids were completely embedded in the OCT compound, transferred to an embedding mold, and stored in a −80°C freezer. The sections (10–20 μm thick) were removed from the freezer, dried at room temperature, and treated with 5% bovine serum albumin (BSA) for 1 hour. Then, the sections were incubated overnight at 4°C with various combinations of primary antibodies specific for the following proteins: SOX2 (CST, 1:300), Phospho‐vimentin (PVIM; MBL International, 1:250, D076‐3 S), TUJ1 (CST, 1:200, 5568), TBR1 (Abcam, 1:300, ab31940), TBR2 (Millipore, 1:100, AB15894), CTIP2 (Abcam, 1:100, ab18465), SATB2 (Abcam, 1:100, ab92446), GFAP (CST, 1:300, 3670 S), GFAPδ (EMD Millipore, 1:500, AB9598), Nestin (CST, 1:200, 4760 S), αB‐Crystallin (Abcam, 1:200, ab76467), Ki‐67 (CST, 1:400, 9129 S), caspase‐3 (Abcam, 1:300, ab32351), caspase‐9 (Abcam, 1:300, ab202068), PDGFRα (Santa Cruz, 1:200, ab61219), Olig2 (Abcam, 1:100, ab109186), O4 (Sigma‐Aldrich, 1:100, MAB1326), myelin basic protein (MBP, CST, 1:100, ab62631), PERK (CST, 1:100, 5683 S), CHOP (CST, 1:400, 2895 S), ATF4 (CST, 1:100, 11815 S), ATF6 (Abcam, 1:100, ab122897), XBP1 (Abcam, 1:100, ab37152), and GADD34 (Abcam, 1:100, ab236516). The slices were washed with phosphate‐buffered solution (PBS) three times and were incubated with Alexa Fluor 568‐conjugated donkey anti‐rabbit IgG (Life Technologies, A11011), Alexa Fluor 488‐conjugated donkey anti‐mouse IgG (Life Technologies, A11001), and Alexa Fluor 647‐conjugated donkey anti‐chicken IgG (Bioss, A104097728) as secondary antibodies for 1 h in the dark. The slices were washed with PBS three times and stained with DAPI (Southern Biotech, 0100‐20). PermaFluor was added, and coverslips were placed on the slices, and the slices were stored at 2–8°C. Images were acquired using a microscope (Fv10‐ASW, Olympus). Image J (imagej.nih.gov/ij/) software was used to measure the area of fluorescence expression of positive cells.

### 2.4 Western blot analysis

RIPA protein lysis buffer (Applygen) was used to prepare lysates from organoids. Proteins and protein ladder (10–180 kDa, Thermo Scientific, 26616) in the samples were separated by 8%–15% sodium dodecyl sulfate‐polyacrylamide gel electrophoresis and transferred to nitrocellulose membranes. The membranes were blocked with a solution containing 5% skimmed milk powder solution, and the protein on the membrane was incubated overnight at 4°C with primary antibodies for β‐actin (Santa Cruz Biotechnology, 1:1000, sc‐47778), GFAP (CST, 1:1000), GFAPδ (EMD Millipore, 1:1000), and caspase‐9 (Abcam, 1:1000). The secondary antibodies used were HRP‐conjugated polyclonal sheep anti‐rabbit (ZSGB‐Bio, 1:1000, ZB‐2301) or anti‐mouse IgG (ZSGB‐Bio, 1:1000, ZB‐2305). Protein–antibody complexes were detected and analyzed using an enhanced chemiluminescence detection kit (Millipore, 2106001).

### 2.5 Transmission electron microscope

Cerebral organoids were collected and transferred to a 50 ml centrifuge tube, washed three times with PBS, added with 2.5% glutaraldehyde fixative, and fixed at 4°C overnight, then fixed with 1% osmium tetroxide for 90 min, dehydrated with ethanol, and embedded in Epon. Brain organoids were prepared into 70 nm thick sections using an ultramicrotome. It was then stained with uranyl acetate and lead citrate for 10 and 12 min, respectively. Finally, the slices were observed and analyzed by transmission electron microscope. G‐ratio refers to the ratio of the diameter of the axon in the myelin sheath to the total outer diameter, which is widely used to evaluate the structure and function of the myelin sheath. Myelin thickness and G‐ratio were calculated by Image J.

### 2.6 Statistical analysis

Statistical analysis was performed by SPSS v26.0 and GraphPad Prism v9.0. All the variables were analyzed by Kolmogorov–Smirnov test to check the normal distribution of the data, while F‐test was used to check homogeneity of variances. The data were presented as mean ± standard error (SEM). For two or three groups of normally distributed data, using two‐tailed t‐test or one‐way ANOVA; otherwise, Mann–Whitney U and Kruskal‐Wilcoxon test were performed. *ns* meant no statistical significance, *p* < 0.05 was considered statistically significant.

## 3 RESULTS

### 3.1 eIF2B mutation resulted in smaller size in cerebral organoids

**FIGURE 2:**
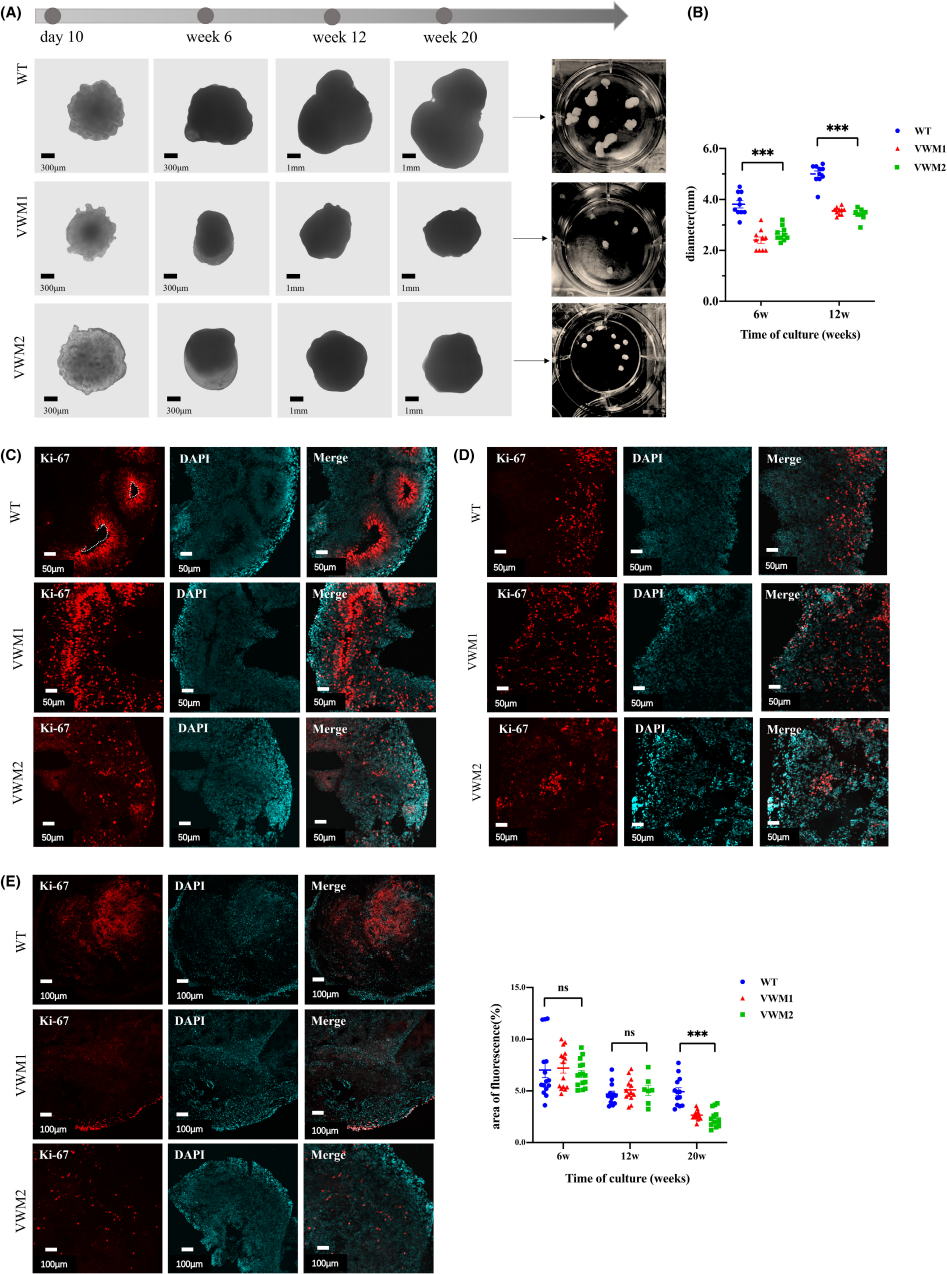
eIF2B mutation resulted in smaller size and showed no difference in proliferation in the early–mid‐stage, but decreased proliferation in later stage in cerebral organoids. (A) Bright‐field images under the microscope of the organoids at different days of maturation. Magnification, scale bar 300 μm and 1 mm. (B) Quantification of the diameter (mm) throughout the entire course of the brain organoid development. Data were presented as mean ± SEM values, *n* = 10 in each group (numbers were listed within each bar) with three independent experiments; one‐way ANOVA analysis for time point. (C) Immunofluorescence staining for cell proliferation markers Ki‐67(red) localize along the ventricle (enclosed by white dotted line), and nuclear staining (DAPI, blue) at Week 6. Scale bars 50 μm. (D) Immunofluorescence staining for Ki‐67(red), and nuclear staining (DAPI, blue) at Week 12. Scale bars 50 μm. (E) Immunofluorescence staining for Ki‐67(red) and nuclear staining (DAPI, blue) at Week 20. Scale bars 100 μm. Changes in cell proliferation quantified by area of fluorescence of Ki‐67, *n* = 12–15 in each group (numbers were listed within each bar) with three independent experiments, one‐way ANOVA, and Kruskal–Wallis ANOVA analysis (Week 6, *p* = 0.767; Week 12, *p* = 0.624; Week 20, *p* = 0.000). Data were presented as mean ± SEM values. ns: *p* > 0.05, ***: *p* < 0.001.

We followed the protocol published by Lancaster et al^11^ to generate cerebral organoids. Equal numbers of dissociated single cells were seeded to form EBs (Days 0–7). After the EBs were embedded in matrigel, they displayed an expanded epithelium after Day 7 and markedly increased in size after 28 days, and the size reached the peak (about 4–6 mm) after 12 weeks of culture. In general, the extended epithelium formation of mutant brain organoids was delayed relative to WT after Day 10 (Figure 2A). The brain organoids were observed under microscope and measured the maximum diameters. The statistical results showed that the sizes of mutant brain organoids were 2.40 ± 0.41 (mm) and 2.65 ± 0.28 (mm), which were significantly smaller than 3.8 ± 0.45 (mm) of WT at Week 6 (Figure 2B, *F* = 37.885, *p* = 0.000). The sizes of mutant brain organoids were 3.55 ± 0.15 (mm) and 3.42 ± 0.21 (mm), which were still significantly smaller than the 5.00 ± 0.38 (mm) of WT (Figure 2B, *F* = 107.012, *p* = 0.000) at Week 12.

### 3.2 Decreased proliferation in later stage of mutant brain organoids

To determine whether the smaller size of mutant brain organoids was due to reduced cell proliferation, cell proliferation quantified by fluorescence of Ki‐67 was assessed. At Week 6, Ki‐67‐positive proliferative progenitor cells were observed located along the ventricle area (enclosed by the dotted line, Figure 2C). Ki‐67‐positive cell proliferation decreased at Week 12 (Figure 2D) and Week 20 (Figure 2E) in both WT and mutant brain organoids. Compared with WT, there was no significant difference in Ki‐67‐positive cells in mutants at Week 6 and Week 12 (Figure 2E, Week 6: *H* = 0.531, *p* = 0.767; Week 12: *F* = 0.479, *p* = 0.624). However, at Week 20, the proportion of Ki‐67‐positive cells in the mutant brain was significantly lower than in WT (Figure 2E, *H* = 22.282, *p* = 0.000). The results suggested that eIF2B mutation has little effect on cell proliferation in the early stage, but may cause limited proliferation in the later stage.

### 3.3 Apoptosis gradually increased from the early stage and was more pronounced in mutant brain organoids

**FIGURE 3:**
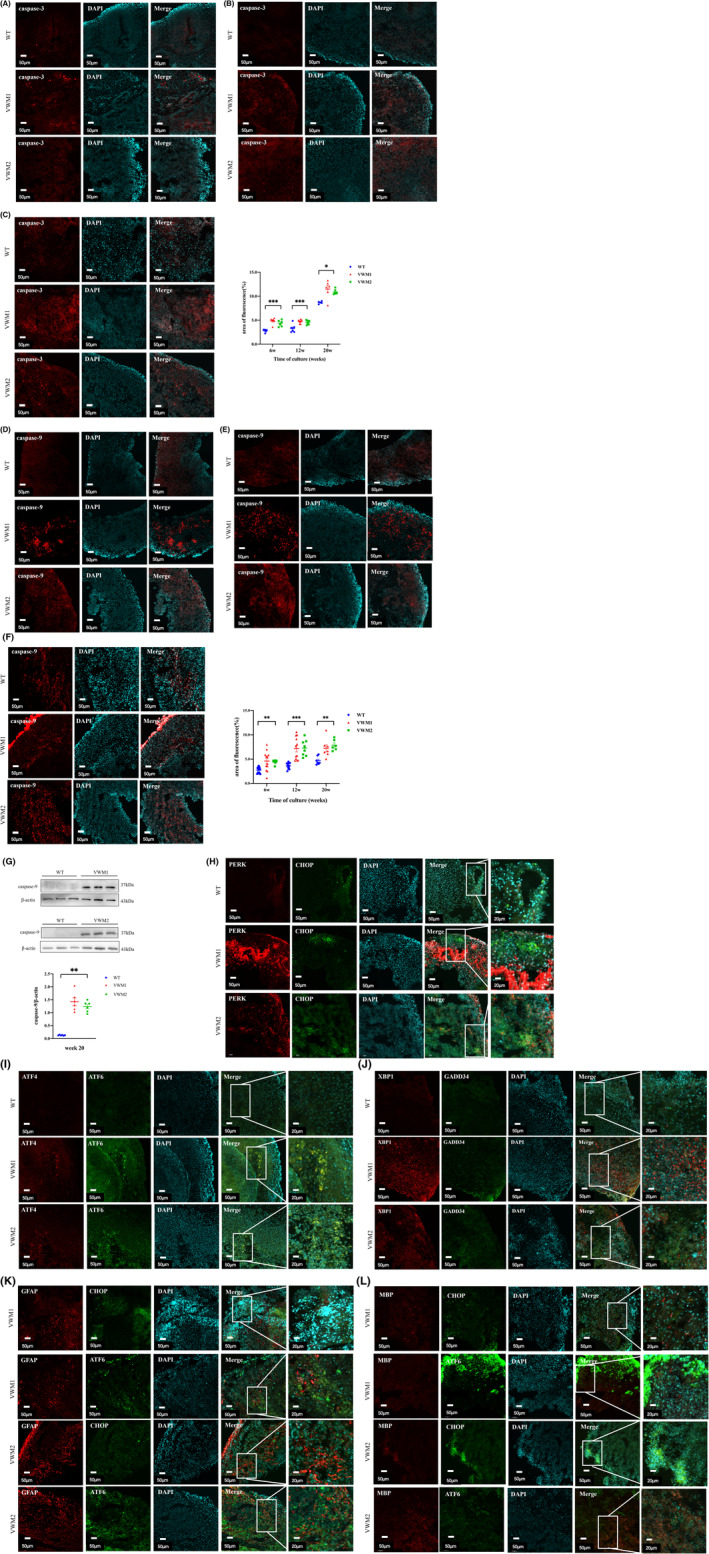
eIF2B mutations led to gradually increased apoptosis of cerebral organoids and UPR signaling pathway overactivation in cerebral organoids. (A) Immunofluorescence staining for cell apoptosis marker caspase‐3(red) and nuclear staining (DAPI, blue) at Week 6. Scale bars 50 μm. (B) Immunofluorescence staining for caspase‐3(red) and nuclear staining (DAPI, blue) at Week 12. Scale bars 50 μm. (C) Immunofluorescence staining for caspase‐3(red) and nuclear staining (DAPI, blue) at Week 20. Scale bars 50 μm. Changes in cell apoptosis quantified by area of fluorescence of caspase‐3, *n* = 5–7 in each group (numbers were listed within each bar) with three independent experiments, one‐way ANOVA analysis (Week 6, *p* = 0.000; Week 12, *p* = 0.000; Week 20, *p* = 0.021). (D) Immunofluorescence staining for cell apoptosis marker caspase‐9(red) and nuclear staining (DAPI, blue) at Week 6. Scale bars 50 μm. (E) Immunofluorescence staining for caspase‐9(red) and nuclear staining (DAPI, blue) at Week 12. Scale bars 50 μm. (F) Immunofluorescence staining for caspase‐9(red) and nuclear staining (DAPI, blue) at Week 20. Scale bars 50 μm. Changes in cell apoptosis quantified by area of fluorescence of caspase‐9, *n* = 6–12 in each group (numbers were listed within each bar) with three independent experiments, one‐way ANOVA, and Kruskal–Wallis ANOVA analysis (Week 6, *p* = 0.004; Week 12, *p* = 0.000; Week 20, *p* = 0.001). (G) Western blotting and qualification of the level of caspase‐9 in cerebral organoids with three independent experiments at Week 20, *n* = 6 in each group (numbers were listed within each bar) with three independent experiments, Kruskal–Wallis analysis. (H) Immunofluorescence staining for PERK(red), CHOP(green), and nuclear staining (DAPI, blue) at Week 20. Scale bars 50 μm. (I) Immunofluorescence staining for ATF4(red), ATF6(green), and nuclear staining (DAPI, blue) at Week 20. Scale bars 50 μm. (J) Immunofluorescence staining for XBP1(red), GADD34(green), and nuclear staining (DAPI, blue) at Week 20. Scale bars 50 μm. (K) Immunofluorescence staining for marker of astrocytes (GFAP, red) and marker of UPR signaling pathway (CHOP, or ATF6, green), and nuclear staining (DAPI, blue) at Week 20. Scale bars 50 μm. (L) Immunofluorescence staining for marker of oligodendrocytes (MBP, red) and marker of UPR signaling pathway (CHOP, or ATF6, green) and nuclear staining (DAPI, blue) at Week 20. Scale bars 50 μm. Data were presented as mean ± SEM values. *: denotes *p* < 0.05, **: denotes *p* < 0.01, ***: denotes *p* < 0.001.

It can be seen from above that there was no significant difference in cell proliferation between mutant and WT brain organoids at Weeks 6 and 12, suggesting that there might be other reasons affecting the size in early stage. Therefore, cell apoptosis was further observe by examining the expression of caspases. Caspases, cysteine protease, play a crucial role in the execution of apoptosis, which is the cascade reaction of caspase activation and apoptotic protease. Pro‐apoptotic caspases mainly include caspase‐2, caspase‐8, caspase‐9, caspase‐10, caspase‐3, caspase‐6, and caspase‐7, which efficiently cleave intracellular proteins in the way of cascade amplification and ultimately lead to cell apoptosis, in which caspase‐2, caspase‐8, caspase‐9, and caspase‐10 participate in the initiation of cell apoptosis as initiators, and caspase‐3, caspase‐6 and caspase‐7 act as effectors. In this experiment, caspase‐3 and caspase‐9 staining were used to evaluate the apoptosis level in brain organoids. With the extension of culture time, the proportions of caspase‐3‐ (Figure 3A–C) and caspase‐9 (Figure 3D–F)‐positive cells were increasing in both mutant and WT brain organoids, suggesting that there was a certain degree of apoptosis in organoids due to the deficiency of central nutrients and oxygen in the process of culture in vitro. The results showed that the proportions of caspase‐3‐ and caspase‐9‐positive cells in mutant brain organoids were significantly higher than that in WT from Week 6 to Week 20 (Figure 3C, caspase‐3, Week 6: *F* = 21.573, *p* = 0.000; Week 12: *F* = 12.888, *p* = 0.000; Week 20: *F* = 7.680, *p* = 0.021; Figure 3F, caspase‐9, Week 6: *H* = 11.288, *p* = 0.004; Week 12: *H* = 21.877, *p* = 0.000; Week 20: *F* = 10.183, *p* = 0.001;), with no significant difference between VWM1 and VWM2 (caspase‐3: *p*_6w_ = 0.136, *p*_12w_ = 0.563, *p*_20w_ = 0.391; caspase‐9: *p*_6w_ = 0.748, *p*_12w_ = 0.808, *p*_20w_ = 0.404). In addition, WB results also confirmed the increased expression of caspase‐9 in mutant brain organoids compared with WT (Figure 3G, *H* = 11.673, *p* = 0.003).

The results suggested that compared with WT, eIF2B mutant brain organoids had little effect on cell proliferation during early development and limited cell proliferation in the later stage. However, increased apoptosis of eIF2B mutant brain organoids was observed throughout the whole developmental process, suggesting that apoptosis was possibly the main factor for the smaller size of mutant brain organoids in the early stage, while the size difference in the later stage was the combined effect of restricted proliferation and increased apoptosis.

### 3.4 Overactivation of the UPR signaling pathway in mutant brain organoids

Abnormal activation of three UPR (unfolded protein response)‐related signaling pathways: PERK, ATF6, and IRE1 pathways were found in the brain tissue samples of VWM patients and mouse models.^12^, ^13^ Therefore, we examined the UPR signaling pathways molecules expression in brain organoids, which might be involved in increased apoptosis. As shown in Figure 3H–J, three UPR signaling pathways molecules (PERK, CHOP, ATF4, ATF6, XBP1, and GADD34) were expressed in mutant brain organoids at Week 20 compared with WT, suggesting overactivation of the UPR signaling pathways in mutant brain organoids. It is known that overactivation of the UPR signaling pathway leads to increased apoptosis, which may partially explain the increase in cell apoptosis in mutant brain organoids. In addition, UPR signaling pathways (CHOP and ATF6) were overactivated in mainly astrocytes and oligodendrocytes in mutant brain organoids (Figure 3K,L).

### 3.5 Delayed early neuronal development, but normal superficial neuronal differentiation in later stage of mutant brain organoids

**FIGURE 4:**
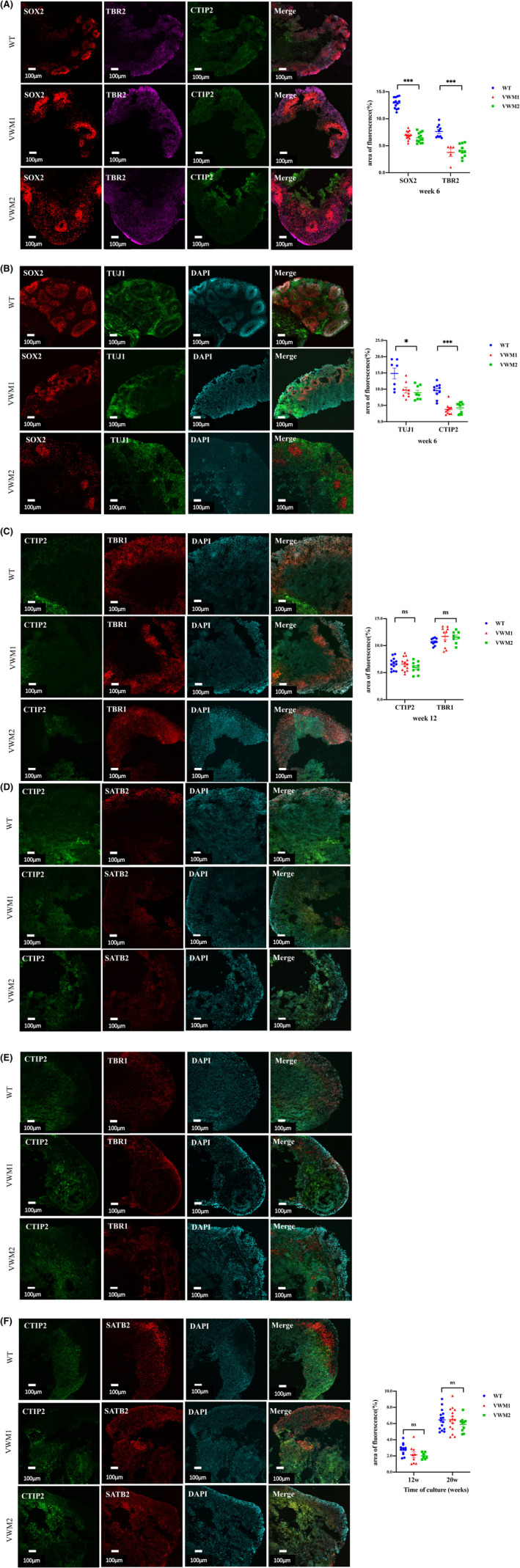
eIF2B mutation resulted in a delay in early neuronal development and normal superficial neuronal differentiation in later stage in cerebral organoids. (A) Immunofluorescence staining for marker of NSCs (SOX2, red), IPs (TBR2, purple), and cortical plate neurons (CTIP2, green) at Week 6. Scale bars 100 μm. *n* = 6–13 organoids (numbers were listed within each bar) from three iPSC lines derived from three individuals, one‐way ANOVA, and Kruskal–Wallis ANOVA analysis. Change in NSCs quantified by area of fluorescence of SOX2, *n* = 6–13 in each group (numbers were listed within each bar) with three independent experiments, one‐way ANOVA analysis (*p* = 0.000). Change in IPs quantified by area of fluorescence of TBR2, *n* = 6–13 in each group (numbers were listed within each bar) with three independent experiments, one‐way ANOVA analysis (*p* = 0.000). (B) Immunofluorescence staining for markers of NSCs (SOX2, red), immature neuron (TUJ1, green), and nuclear staining (DAPI, blue) at Week 6. Scale bars 100 μm. Change in immature neurons quantified by area of fluorescence of TUJ1, *n* = 6–13 in each group (numbers were listed within each bar) with three independent experiments, one‐way ANOVA analysis (*p* = 0.021). Change in cortical plate neurons quantified by area of fluorescence of CTIP2, *n* = 6–13 in each group (numbers were listed within each bar) with three independent experiments, one‐way ANOVA analysis (*p* = 0.000). (C) Immunofluorescence staining for cortical plate neuron (CTIP2, green), deep‐layer neurons (TBR1, red), and nuclear staining (DAPI, blue) at Week 12. Scale bars 50 μm. Change in cortical plate neurons quantified by area of fluorescence of CTIP2, *n* = 6–13 in each group (numbers were listed within each bar) with three independent experiments, one‐way ANOVA analysis (*p* = 0.350). Change in deep‐layer neurons quantified by area of fluorescence of TBR1, *n* = 6–13 in each group (numbers were listed within each bar) with three independent experiments, one‐way ANOVA analysis (*p* = 0.220). (D) Immunofluorescence staining for cortical plate neuron (CTIP2, green), superficial neurons (SATB2, red), and nuclear staining (DAPI, blue) at Week 12. Scale bars 50 μm. (E) Immunofluorescence staining for cortical plate neuron (CTIP2, green), deep‐layer neurons (TBR1, red), and nuclear staining (DAPI, blue) at Week 20. Scale bars 50 μm. (F) Immunofluorescence staining for cortical plate neuron (CTIP2, green), superficial neurons (SATB2, red), and nuclear staining (DAPI, blue) at Week 20. Scale bars 50 μm. Changes in superficial neurons quantified by area of fluorescence of SATB2, *n* = 6–13 in each group (numbers were listed within each bar) with three independent experiments, one‐way ANOVA analysis (Week 12: *p* = 0.060; Week 20: *p* = 0.481). NSC: neural stem cell; IP: intermediate progenitor cells. Data were presented as the mean ± SEM values, ns: *p* > 0.05, **: *p* < 0.01, ***: *p* < 0.001.

At Week 6, SOX2‐positive neural stem cells (NSCs) were radially distributed around the pseudoventricle with TUJ1‐positive immature neurons adjacent, which together formed the ventricle zone (VZ). Intermediate progenitor cells (IPs, TBR2‐positive) migrated from the ventricular region to the center and formed the subventricular zone (SVZ), while the cells in the inner and outer center of the progenitor cells express cortical plate (CP) markers (CTIP2), as shown in Figure 4A,B. Therefore, the early brain organ mimics the VZ (SOX2‐positive NSC), SVZ (TBR2‐positive IP), and CP (CTIP2‐positive cortical plate neurons) during the development of the human brain. Compared with WT, mutant brain organoids showed fewer rose‐like structures and more disordered cell arrangements, the proportions of SOX2‐positive neural stem cells and TBR2‐positive intermediate progenitor cells (Figure 4A, SOX2: *F* = 200.840, *p* = 0.000; TBR2: *F* = 24.631, *p* = 0.000). The proportion of immature neurons and cortical plate neurons was lower than that of WT (Figure 4B, TUJ1: *H* = 7.734, *p* = 0.021; CTIP2: *F* = 29.467, *p* = 0.000).

At Week 12, the brain organoids were further mature, SOX2‐positive apical NSCs were confined to the VZ region, and expanded dorsally to further differentiate into neurons. TUJ1‐positive immature neurons differentiated into deeper layer (TBR1‐positive) and superficial layer (SATB2‐positive) neurons. It was found that the proportion of CTIP2‐positive cortical plate neurons and TBR1‐positive deeper layer neurons in mutant brain organoids was no statistically different compared with WT (Figure 4C, *F* = 1.086, *p* = 0.350; *F* = 3.033, *p* = 0.220). In addition, as shown in Figure 4D, the proportion of SATB2‐positive superficial neurons in both WT and eIF2B mutant brain organoids was still little and there was no statistical difference (Figure 4F, *F* = 3.149, *p* = 0.060).

At Week 20, with the prolongation of culture time, the brain organoids continued to proliferate and mature, as shown in Figure 4E,F, SATB2‐positive superficial neurons increased greatly, while CTIP2‐positive cortical plate neurons and TBR1‐positive deeper layer neurons decreased. The proportion of SATB2‐positive superficial neurons increased significantly, and no statistical difference was found between WT and mutant (Figure 4F, *F* = 0.745, *p* = 0.481).

The results showed that compared with WT, during early (Week 6) cortical development, eIF2B mutation resulted in fewer neural stem cells, intermediate progenitor cells, and neuronal cells in brain organoids. When further cultured and differentiated to mid‐stage (Week 12), the proportions of CTIP2‐positive and TBR1‐positive deeper neurons in eIF2B mutant brain organoids tended to be consistent. There was no difference in the proportion of SATB‐positive superficial neurons in the cortex when they continued to proliferate and mature to the later stage (Week 20). It is suggested that eIF2B mutation affects the development of neural stem cells in the early stage, but there is no difference in the mature neurons in the middle and later stages, and this may be explained by a complex temporal and spatial regulation mechanism.

### 3.6 Immature astrocytes with increased expression of GFAPδ, nestin, and αB‐crystallin in mutant brain organoids

**FIGURE 5:**
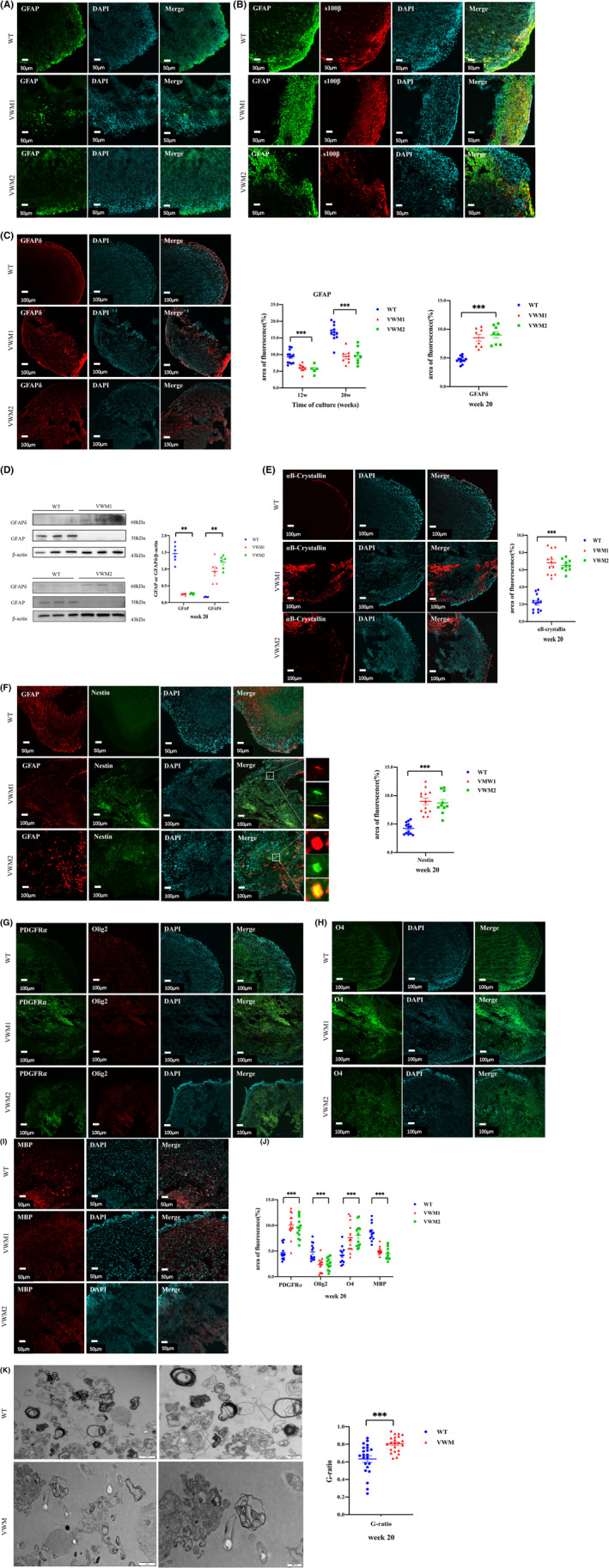
eIF2B mutation resulted in impaired differentiation of astrocytes and oligodendrocytes. (A) Immunofluorescence staining for marker of astrocytes (GFAP, green) and nuclear staining (DAPI, blue) at Week 12. Scale bars 50 μm. (B) Immunofluorescence staining for markers of astrocytes (GFAP, green and s100β, red) and nuclear staining (DAPI, blue) at Week 20. Scale bars 50 μm. Changes in astrocytes quantified by area of fluorescence of GFAP, *n* = 8–12 in each group with three independent experiments, one‐way ANOVA analysis (Week 12: *p* = 0.000; Week 20: *p* = 0.000). (C) Immunofluorescence staining for the abnormal isomer GFAPδ(red) of astrocytes and nuclear staining (DAPI, blue) at Week 20. Scale bars 50 μm. Change in abnormal astrocytes quantified by area of fluorescence of GFAPδ, *n* = 8–12 in each group with three independent experiments, one‐way ANOVA analysis (*p* = 0.000). (D) Western blotting and qualification of the level of GFAP and GFAPδ in cerebral organoids with three independent experiments at Week 20, *n* = 6 in each group (numbers were listed within each bar) with three independent experiments, Kruskal–Wallis analysis. (E) Immunofluorescence staining for the abnormal marker of astrocytes αB‐crystallin (red) and nuclear staining (DAPI, blue) at Week 20. Scale bars 100 μm. Change in abnormal astrocytes quantified by area of fluorescence of αB‐crystallin, *n* = 10–13 in each group with three independent experiments, one‐way ANOVA analysis (*p* = 0.000). (F) Immunofluorescence staining for the immature marker nestin(green) of astrocytes and nuclear staining (DAPI, blue) at Week 20. Scale bars 50 μm. Change in abnormal astrocytes quantified by area of fluorescence of nestin, *n* = 10–13 in each group with three independent experiments, one‐way ANOVA analysis (*p* = 0.000). (G) Immunofluorescence staining for the marker of OPCs (PDGFRα, green), mature oligodendrocytes (Olig2, red), and nuclear staining (DAPI, blue) at Week 20. Scale bars 100 μm. (H) Immunofluorescence staining for the immature marker of oligodendrocytes (O4, green) and nuclear staining (DAPI, blue) at Week 20. Scale bars 100 μm. (I) Immunofluorescence staining for the mature marker of oligodendrocytes (MBP, red), and nuclear staining (DAPI, blue) at Week 20. Scale bars 100 μm. (J) Change in OPCs quantified by area of fluorescence of PDGFRα, *n* = 13 in each group with three independent experiments, one‐way ANOVA analysis (*p* = 0.000). Changes in oligodendrocytes quantified by area of fluorescence of Olig2, O4, and MBP, *n* = 12–13 in each group with three independent experiments, one‐way ANOVA analysis (Olig2: *p* = 0.000; O4: *p* = 0.000; MBP: *p* = 0.000). (K) Transmission electron microscopy showed the myelin sheath. Scale bars 1 μm (left), 500 nm (right). Quantification of G‐ratio at Week 20. Mann–Whitney U‐test analysis (*p* = 0.000). OPC: oligodendrocyte precursor cells. Data were presented as mean ± SEM values, **: *p* < 0.01, ***: *p* < 0.001.

GFAP is mainly expressed in astrocytes and is considered a marker of astrocyte maturation. As shown in Figure 5A. From Week 12, the GFAP‐positive astrocytes gradually increased with the extension of culture. At Weeks 12 and 20, the proportion of GFAP‐positive mature astrocytes in mutant brain organoids was significantly lower than that in WT (Figure 5B, Week 12: *F* = 15.691, *p* = 0.000; Week 20: *F* = 29.583, *p* = 0.000), with no significant difference between VWM1 and VWM2 (*p* = 0.615, *p* = 0.826). Western blot (WB) also confirmed that the expression of GFAP in mutants was significantly reduced compared with WT (Figure 5D, *H* = 11.863, *p* = 0.003).

GFAPδ is a GFAP protein allotype encoded by a splicing variant of the *GFAP* gene, which is considered to have a negative effect on GFAP filament formation. As an abnormal isomer, GFAPδ usually does not increase during the proliferation and maturation of astrocytes. However, the proportion of GFAPδ in astrocytes of mutant brain organoids was much higher than WT (Figure 5C, *F* = 35.739, *p* = 0.000), and the expression was significantly increased confirmed by WB (Figure 5D, *H* = 11.954, *p* = 0.003) at Week 20. In addition, αB‐crystallin, a well‐defined member of the small heat shock protein family, is expressed mainly in astrocytes and acts as a chaperone to bind unfolded proteins to inhibit the aggregation like GFAPδ. At Week 20, the proportion of αB‐crystallin‐positive cells in mutant brain organoids was significantly higher than that in WT (Figure 5E, *H* = 23.877, *p* = 0.000).

Nestin is an intermediate silk protein, mainly expressed in undifferentiated stem cells. When stem cells differentiate into neurons and glial cells, the expression of nestin would be reduced or even not expressed. At Week 20, the proportion of nestin‐positive GFAP‐positive astrocytes in mutant brain organoids was significantly higher than that of WT (Figure 5F, *H* = 23.921, *p* = 0.000).

### 3.7 Increased oligodendrocyte progenitor cells, decreased mature oligodendrocytes, and sparse myelin in mutant cerebral organoids

The brain organoids continue to develop until glial cells appeared in the later stage. At Week 20, it was observed the number of oligodendrocyte progenitor cells (OPCs) and oligodendrocytes in brain organoids (Figure 5G–I). PDGFRα is a marker of oligodendrocyte progenitor cells (OPCs), and O4 is the earliest recognized lineage‐specific marker of oligodendrocyte as a marker of immature oligodendrocyte. MBP is a structural component of myelin, which is expressed only by myelin glia and is a marker of myelin function in oligodendrocyte maturation. Olig2 is expressed in mature oligodendrocytes. It was found that compared with WT, the proportion of PDGFRα‐positive OPCs in mutant brain organoids significantly increased (Figure 5J, *F* = 28.627, *p* = 0.000), and the proportion of O4‐positive immature oligodendrocytes were significantly more (Figure 5J, *F* = 12.067, *p* = 0.000), while the proportions of Olig2‐positive and MBP‐positive oligodendrocytes in mutants were significantly lower compared with WT (Figure 5J, Olig2: *F* = 12.648, *p* = 0.000; MBP: *F* = 36.059, *p* = 0.000) at Week 20.

In addition, a number of myelin‐like structures were observed at Week 20 (Figure 5K). Myelin‐like structures in WT brain organoids were helical or concentrically coiled, whereas myelin sheaths were sparse and thinner in mutant brain organoids. G‐ratio is the ratio of the diameter of the axon within the myelin sheath to the total outer diameter and is used to evaluate the structure and function of the myelin sheath. Statistical results showed that the G‐ratio ratio of mutant brain organoids was significantly greater than that of WT (Figure 5K, *Z* = 3.615, *p* = 0.000).

## 4 DISCUSSION

In this study, we established *EIF2B4* and *EIF2B5* mutant brain organoids for the first time and observed the dynamic developmental process. The organoids spontaneously self‐pattern and self‐organize into distinct brain regions without directing a specific identity. Several studies have found that organoids replicate brain development and generated a wide diversity of cells that share transcriptomic spectroscopy with the early fetal neocortex.^14^, ^15^, ^16^ Organoids show spatial organization of proliferative progenitors into VZ and SVZ‐like regions, followed by neurons with primitive inside‐out layering into deep deep‐layer neurons followed by upper‐layer neurons that migrate more superficially. Long‐term cultured organoids reveal GFAP‐expressing astrocytes and oligodendrocyte lineage cells (OPC and OL).

An interesting finding in our study was that the volume of mutant brain organoids was significantly smaller than the wild type from early stage (Week 6). However, most patients with VWM had normal head circumference. Microcephaly was only reported in patients with congenital VWM. A total of 10 patients of congenital type have been reported so far,^6^, ^17^, ^18^ which could be afflicted in the fetus, manifested as severe intrauterine growth retardation, oligohydramnios and decreased fetal movement, encephalopathy symptoms, and maybe with multiple system involvement. There was currently no genotypic explanation for the early onset in these patients. Due to the limitations of the 3D culture system, oxygen and nutrients transported to the brain organoids are insufficient to provide various nerve cells with high metabolic requirements, and the lack of a vascular system in brain organoids leads to continuous cell apoptosis and cell death in the central region. Organoids are under metabolic stress and oxidative stress regardless of the method of culture, cell line, or culture time employed.^19^ In our study, under the same culture conditions, compared with wild‐type, apoptosis was significantly increased in mutant brain organoids, and the level of apoptosis was increasingly higher with the extension of culture time, suggesting that eIF2B mutation leads to increased susceptibility to stress.

eIF2B is an important factor in the translation of mRNA into protein and regulates the rate of protein synthesis. A central mechanism in a cellular stress response is the inhibition of protein synthesis, known as unfolded protein response, mainly mediated by protein kinase R‐like endoplasmic reticulum kinase (PERK), activating transcription factor 6 (ATF6), and inositol requiring enzyme 1 (IRE1).^13^ This process aims to reduce the accumulation of denatured and misfolded proteins and escape translation inhibition through the transcription of mRNA with a special open reading frame (ORF), thus preserving cellular energy to enhance cell survival under stress. In addition, different cellular stresses would lead to eIF2α phosphorylation, which then acts as a competitive inhibitor of eIF2B. eIF2B also participates in the UPR by coordinating the integrated stress response (ISR), involving four kinases: general control nonderepressible 2 (GCN2), protein kinase RNA activated (PKR), PERK, and heme‐regulated inhibitor kinase (HRI).^20^ However, if the cellular stress state remains uncorrected for a long time and the unfolded protein response is overactivated and could not be recovered over a long period of time, it may lead to the logical deterioration and possible multi‐organ dysfunction, which may be the possible cause of exacerbation of VWM. Previous studies have demonstrated that higher susceptibility to endoplasmic reticulum stress (ERS) and upregulations of unfolded protein response (UPR) in VWM.^12^, ^13^, ^21^ We confirmed that the UPR signaling pathway was activated in mutant brain organoids at Week 20. Therefore, we believe that in the culture system with relative metabolic stress, due to the increased susceptibility to stress, the apoptosis of mutant brain organoids is abnormally increased, resulting in a decrease in size. This may suggest that the microcephaly in congenital patients may be not only caused by eIF2B mutations, but combined with premature exposure to the stressful environment such as hypoxia‐ischemia in the uterine.

In normal brain organoids, GFAP‐positive astrocytes usually begin to appear after Day 100 of culture.^22^ In eIF2B mutant brain organoids, we also observed GFAPα‐positive astrocytes at 12 weeks, but in far fewer numbers than the wild type, and more cells expressing the nestin (maker of undifferentiated stem cells) and GFAPδ isoforms. Astrocytes are differentiated from intermediate progenitor cells, which are generated by radial glial cells. During this process, transcriptional factors and related signaling pathways activate the expression of the *GFAP* gene, which is a marker of terminally differentiated astrocytes. However, the procedure of specification programming is not fully understood. It has been suggested that the interactions of JAK–STAT, BMP‐Smad, and Notch signaling pathways and transcription factors SOX9 and NFIA are related to their programmed differentiation,^23^, ^24^ which indicates us to further analyze the gene transcriptome changes during the dynamic development of astrocytes by single‐cell transcriptome analysis of brain organoids. The *GFAP* gene products mainly contain three isomers (α, δ, and κ). GFAPα is the most abundantly expressed in the brain, forming a dense cytoskeletal network. The elevated expression of GFAPδ leads to the disturbance of the interfilament network in astrocytes, resulting in morphological abnormalities and restricted reactive gliosis.^25^ We also found overexpression of αB‐crystallin in mutant brain organoids, which is a small heat shock protein that binds and stabilizes unstable conformational isomers of proteins. Overexpression of αB‐crystallin in VWM is probably a protective response that protects astrocytes from GFAPδ accumulation. However, the persistence of protective stress response may lead to a pathological state and abnormal morphology of astrocytes. It was found that morphological abnormality was only observed in GFAPδ‐positive astrocytes in VWM mice.^26^

The appearance of OPCs and oligodendrocytes in mutant brain organoids were observed at Week 20, and the timing was similar to normal brain organoids.^22^ We found an increase in PDGFRα‐positive OPCs, and an increased expression of O4 (immature marker of oligodendrocytes), while the expression of Olig2 and MBP decreased with thin myelin in mutant brain organoids. The findings were similar to the pathology from autopsy sample. Previous studies found that the differentiation and development of eIF2B mutant oligodendrocytes cultured in vitro were normal.^10^, ^27^ However, in vitro co‐culture system, mutant astrocytes inhibited the maturation of OPC.^26^ These studies indicated that astrocytes were probably central in VWM pathomechanisms. We found that UPR was present in both astrocytes and oligodendrocytes in mutant brain organoids. Astrocytes were reported to be resistant to ERS‐induced cell death caused by active UPR signaling.^28^ However, in response to UPR overactivation, astrocytes produced dysregulated inflammation, reduced cytotrophic support, and could transmit ER stress to other cells. Therefore, the abnormality of oligodendrocytes in vivo may be due to the increase in apoptosis on the one hand, and the influence of astrocyte dysfunction on the other hand. This prompts us to further investigate the cross‐talk between astrocytes and oligodendrocytes in brain organoids.

Neurons were relatively spared in the cadaveric brain pathology of VWM patients,^2^ whereas Trimouille et al^6^ found that transient external granulocytes, Purkinje cells, and the inner granulocyte layer of the cerebellum were dysplastic in the fetal cadaveric brain of congenital VWM, which may affect the migration and survival of neurons and resulted in cerebellar dysplasia. These results indicated that eIF2B mutation may have a certain influence on neuronal development. We found that the neural development of mutant organoids roughly followed the timing pattern of initially making deep‐layer (CTIP2^+^), followed by upper‐layer (SATB2^+^) neurons. But in the process of early cortical development, the proportion of neural stem cells, intermediate progenitor cells, and immure neurons in mutant brain organoids were reduced, whereas SATB2‐positive superficial neurons were normal.

There are some limitations in this study. First, only two cases of VWM patient‐derived iPSCs and one control iPSC were used in the experiment, the small sample size may reduce the representability of research results to some extent. Second, due to the lack of vascular networks, the supply of oxygen and nutrients are limited in the core of the organoid in the culture system. Third, the time points selected to observe brain development in this study are not enough to fully reproduce the development of the nervous system. We have found the absence of myelination by observing the formation and structure of myelin sheath, but we have not studied the function of myelin in depth. We would further study the function of myelin in brain organoids in the following studies.

## 5 CONCLUSIONS

In this study, we constructed the first *EIF2B4* and *EIF2B5* mutant brain organoids, which provided a new system for investigating the pathophysiological mechanisms in the dynamic developmental process of VWM. Mutant brain organoids showed smaller size from early stage, which was probably due to increased apoptosis resulting from UPR overactivation. This result may explain the microcephaly in patients with congenital form of VWM. In the early stage of mutant brain organoids, there was a delay in neuronal development. Whereas in the later stage, disturbance in the maturation of astrocyte and oligodendrocyte was prominent, with normal superficial neurons. Combining with the timing of glial cell differentiation, it is suggested that eIF2B mutation affects the development of neural stem cells in the early stage of brain organoids, which in turn influence the development of subsequent glial cells in later stage. Complex temporal and spatial regulation mechanisms for the explanation of the phenomenon need further elucidation.

## AUTHOR CONTRIBUTIONS

JD, JZ, and YW conceptualized the study. JD, JZ, and LZ curated the data. JD and KG contributed to the formal analysis. JD and YW wrote the original draft. JW, YJ, and YW contributed to the review and editing.

## FUNDING INFORMATION

This study was supported by the Program of the Natural Science Foundation of China (82171694 and 81901155), Beijing Natural Science Foundation (L202034), and the National Science and Technology Major Project of the Ministry of Science and Technology of China (grant nos. 2017ZX09304029‐006).

## CONFLICT OF INTEREST

The authors declare that the research was conducted in the absence of any commercial or financial relationships that could be construed as a potential conflict of interest.

## Data Availability

The original contributions presented in the study are included in the article and supplementary material, further inquiries can be directed to the corresponding authors.

## References

[1] Hamilton EMC , van der Lei HDW , Vermeulen G , et al. Natural history of vanishing white matter. Ann Neurol. 2018;84(2):274‐288. 30014503 10.1002/ana.25287 PMC6175238

[2] van der Knaap MS , Pronk JC , Scheper GC . Vanishing white matter disease. Lancet Neurol. 2006;5(5):413‐423. 16632312 10.1016/S1474-4422(06)70440-9

[3] Wu Y , Jiang YW , Qin J , et al. Clinical characteristics of cases with leukoencephalopathy with vanishing white matter. Zhonghua Er Ke Za Zhi. 2007;45(2):115‐120. 17456339

[4] Deng J , Zhou L , Zhang J , et al. Correlation between genotype and age of onset in leukoencephalopathy with vanishing white matter. Front Genet. 2021;12:729777. 34745209 10.3389/fgene.2021.729777 PMC8564072

[5] Bugiani M , Boor I , van Kollenburg B , et al. Defective glial maturation in vanishing white matter disease. J Neuropathol Exp Neurol. 2011;70(1):69‐82. 21157376 10.1097/NEN.0b013e318203ae74 PMC4135437

[6] Trimouille A , Marguet F , Sauvestre F , et al. Foetal onset of EIF2B related disorder in two siblings: cerebellar hypoplasia with absent Bergmann glia and severe hypomyelination. Acta Neuropathol Commun. 2020;8(1):48. 32293553 10.1186/s40478-020-00929-2 PMC7161274

[7] Lancaster MA , Renner M , Martin CA , et al. Cerebral organoids model human brain development and microcephaly. Nature. 2013;501(7467):373‐379. 23995685 10.1038/nature12517 PMC3817409

[8] Di Lullo E , Kriegstein AR . The use of brain organoids to investigate neural development and disease. Nat Rev Neurosci. 2017;18(10):573‐584. 28878372 10.1038/nrn.2017.107 PMC5667942

[9] Sloan SA , Darmanis S , Huber N , et al. Human astrocyte maturation captured in 3D cerebral cortical spheroids derived from pluripotent stem cells. Neuron. 2017;95(4):779‐790.e776. 28817799 10.1016/j.neuron.2017.07.035 PMC5890820

[10] Zhou L , Li P , Chen N , et al. Modeling vanishing white matter disease with patient‐derived induced pluripotent stem cells reveals astrocytic dysfunction. CNS Neurosci Ther. 2019;25(6):759‐771. 30720246 10.1111/cns.13107 PMC6515702

[11] Lancaster MA , Knoblich JA . Generation of cerebral organoids from human pluripotent stem cells. Nat Protoc. 2014;9(10):2329‐2340. 25188634 10.1038/nprot.2014.158 PMC4160653

[12] van Kollenburg B , van Dijk J , Garbern J , et al. Glia‐specific activation of all pathways of the unfolded protein response in vanishing white matter disease. J Neuropathol Exp Neurol. 2006;65(7):707‐715. 16825957 10.1097/01.jnen.0000228201.27539.50

[13] van der Voorn JP , van Kollenburg B , Bertrand G , et al. The unfolded protein response in vanishing white matter disease. J Neuropathol Exp Neurol. 2005;64(9):770‐775. 16141786 10.1097/01.jnen.0000178446.41595.3a

[14] Paşca AM , Sloan SA , Clarke LE , et al. Functional cortical neurons and astrocytes from human pluripotent stem cells in 3D culture. Nat Methods. 2015;12(7):671‐678. 26005811 10.1038/nmeth.3415 PMC4489980

[15] Camp JG , Badsha F , Florio M , et al. Human cerebral organoids recapitulate gene expression programs of fetal neocortex development. Proc Natl Acad Sci USA. 2015;112(51):15672‐15677. 26644564 10.1073/pnas.1520760112 PMC4697386

[16] Pollen AA , Bhaduri A , Andrews MG , et al. Establishing cerebral organoids as models of human‐specific brain evolution. Cell. 2019;176(4):743‐756.e717. 30735633 10.1016/j.cell.2019.01.017 PMC6544371

[17] van der Knaap MS , van Berkel CG , Herms J , et al. eIF2B‐related disorders: antenatal onset and involvement of multiple organs. Am J Hum Genet. 2003;73(5):1199‐1207. 14566705 10.1086/379524 PMC1180499

[18] Song H , Haeri S , Vogel H , van der Knaap M , Van Haren K . Postmortem whole exome sequencing identifies novel EIF2B3 mutation with prenatal phenotype in 2 siblings. J Child Neurol. 2017;32(10):867‐870. 28597716 10.1177/0883073817712588

[19] Bhaduri A , Andrews MG , Mancia Leon W , et al. Cell stress in cortical organoids impairs molecular subtype specification. Nature. 2020;578(7793):142‐148. 31996853 10.1038/s41586-020-1962-0 PMC7433012

[20] Pavitt GD . Regulation of translation initiation factor eIF2B at the hub of the integrated stress response. Wiley Interdiscip Rev RNA. 2018;9(6):e1491. 29989343 10.1002/wrna.1491

[21] Abbink TEM , Wisse LE , Jaku E , et al. Vanishing white matter: deregulated integrated stress response as therapy target. Ann Clin Transl Neurol. 2019;6(8):1407‐1422. 31402619 10.1002/acn3.50826 PMC6689685

[22] Renner M , Lancaster MA , Bian S , et al. Self‐organized developmental patterning and differentiation in cerebral organoids. EMBO J. 2017;36(10):1316‐1329. 28283582 10.15252/embj.201694700 PMC5430225

[23] He F , Ge W , Martinowich K , et al. A positive autoregulatory loop of Jak‐STAT signaling controls the onset of astrogliogenesis. Nat Neurosci. 2005;8(5):616‐625. 15852015 10.1038/nn1440 PMC4222251

[24] Kang P , Lee HK , Glasgow SM , et al. Sox9 and NFIA coordinate a transcriptional regulatory cascade during the initiation of gliogenesis. Neuron. 2012;74(1):79‐94. 22500632 10.1016/j.neuron.2012.01.024 PMC3543821

[25] Bugiani M , Vuong C , Breur M , van der Knaap MS . Vanishing white matter: a leukodystrophy due to astrocytic dysfunction. Brain Pathol. 2018;28(3):408‐421. 29740943 10.1111/bpa.12606 PMC8028328

[26] Dooves S , Bugiani M , Postma NL , et al. Astrocytes are central in the pathomechanisms of vanishing white matter. J Clin Invest. 2016;126(4):1512‐1524. 26974157 10.1172/JCI83908 PMC4811153

[27] Dietrich J , Lacagnina M , Gass D , et al. EIF2B5 mutations compromise GFAP+ astrocyte generation in vanishing white matter leukodystrophy. Nat Med. 2005;11(3):277‐283. 15723074 10.1038/nm1195

[28] Sims SG , Cisney RN , Lipscomb MM , Meares GP . The role of endoplasmic reticulum stress in astrocytes. Glia. 2022;70(1):5‐19. 34462963 10.1002/glia.24082 PMC9292588

